# Impact of a “Chart Closure” Hard Stop Alert on Prescribing for Elevated Blood Pressures Among Patients With Diabetes: Quasi-Experimental Study

**DOI:** 10.2196/16421

**Published:** 2020-04-17

**Authors:** Magaly Ramirez, Kimberly Chen, Robert W Follett, Carol M Mangione, Gerardo Moreno, Douglas S Bell

**Affiliations:** 1 Department of Health Services School of Public Health University of Washington Seattle, WA United States; 2 Clinical Informatics UCLA Health Los Angeles, CA United States; 3 Division of General Internal Medicine and Health Services Research, Department of Medicine David Geffen School of Medicine University of California at Los Angeles Los Angeles, CA United States; 4 Department of Health Policy and Management Fielding School of Public Health University of California at Los Angeles Los Angeles, CA United States; 5 Department of Family Medicine David Geffen School of Medicine University of California at Los Angeles Los Angeles, CA United States

**Keywords:** decision support systems, clinical, diabetes mellitus, hypertension, drug prescriptions

## Abstract

**Background:**

University of California at Los Angeles Health implemented a Best Practice Advisory (BPA) alert for the initiation of an angiotensin-converting enzyme inhibitor (ACEI) or angiotensin-receptor blocker (ARB) for individuals with diabetes. The BPA alert was configured with a “chart closure” hard stop, which demanded a response before closing the chart.

**Objective:**

The aim of the study was to evaluate whether the implementation of the BPA was associated with changes in ACEI and ARB prescribing during primary care encounters for patients with diabetes.

**Methods:**

We defined ACEI and ARB prescribing opportunities as primary care encounters in which the patient had a diabetes diagnosis, elevated blood pressure in recent encounters, no active ACEI or ARB prescription, and no contraindications. We used a multivariate logistic regression model to compare the change in the probability of an ACEI or ARB prescription during opportunity encounters before and after BPA implementation in primary care sites that did (n=30) and did not (n=31) implement the BPA. In an additional subgroup analysis, we compared ACEI and ARB prescribing in BPA implementation sites that had also implemented a pharmacist-led medication management program.

**Results:**

We identified a total of 2438 opportunity encounters across 61 primary care sites. The predicted probability of an ACEI or ARB prescription increased significantly from 11.46% to 22.17% during opportunity encounters in BPA implementation sites after BPA implementation. However, in the subgroup analysis, we only observed a significant improvement in ACEI and ARB prescribing in BPA implementation sites that had also implemented the pharmacist-led program. Overall, the change in the predicted probability of an ACEI or ARB prescription from before to after BPA implementation was significantly greater in BPA implementation sites compared with nonimplementation sites (difference-in-differences of 11.82; *P*<.001).

**Conclusions:**

A BPA with a “chart closure” hard stop is a promising tool for the treatment of patients with comorbid diabetes and hypertension with an ACEI or ARB, especially when implemented within the context of team-based care, wherein clinical pharmacists support the work of primary care providers.

## Introduction

### Background

Given the increasing interest in using health information technology to enhance diabetes care, it is critically important to examine the impact of these interventions on quality of care [1,2]. Clinical decision support (CDS) systems interfaced with electronic health record (EHR) systems can notify a primary care provider (PCP) when there are deviations from the accepted standards of diabetes care. However, there is limited research examining the impact of EHR-based CDS systems on the initiation of antihypertensive therapies for patients with comorbid diabetes and hypertension [3]. It is estimated that 20% to 60% of the patients with diabetes have hypertension [4], yet only 10% to 13% of these patients receive adequate treatment [5-7]. The standards of diabetes care developed by the American Diabetes Association urge the timely treatment of hypertension using an angiotensin-converting enzyme inhibitor (ACEI) or angiotensin-receptor blocker (ARB), as these medications decrease the risk for microvascular and macrovascular complications [8]. The presence and severity of diabetes-related complications are associated with increased health care utilization and costs [9]. Well-trained PCPs are familiar with the recommendation to treat hypertension in patients with diabetes, but sometimes, because of patient complexity or nonadherence, there may be overlooked opportunities when patients could take an ACEI or ARB. Therefore, at University of California at Los Angeles (UCLA) Health, we implemented a CDS system that alerted PCPs of any patient with diabetes who was missing one of these medications and had no contraindications.

EHR-based CDS systems promise to accelerate the adoption of evidence-based care [10,11], but there remains a gap in our knowledge about effective CDS system designs to prompt the initiation of effective therapies in patients with diabetes. In particular, there is an opportunity to study the impact of readily available CDS tools within EHR systems, such as the Best Practice Advisory (BPA) within the Epic EHR system (Epic Systems Corporation, Verona, WI), to prompt PCPs when there is an indication to start a patient with diabetes on an ACEI or ARB. Previous studies have evaluated the impact of BPAs using pre-post study designs, but with no comparison group [12-14]. Some have observed increased compliance with clinical practice guidelines after the implementation of a BPA [12,13], whereas others have observed no significant changes [14]. We are not aware of previous research having comprehensively examined the impact of a BPA using a more rigorous quasi-experimental difference-in-differences design or the impact of a BPA on diabetes care. Rigorous evaluations of electronic CDS tools are needed to understand their impact on quality of care and patient outcomes [15,16].

Between 2014 and 2015, UCLA Health implemented a narrowly targeted BPA within CareConnect—its implementation of the Epic EHR system—which fires alerts to PCPs during primary care encounters when a patient with diabetes has elevated blood pressure readings, is not on an ACEI or ARB, and has no contraindications. Our previous work examining the first eight of the 30 sites that implemented the BPA suggested that the BPA, when coupled with a “chart closure” hard stop, might improve PCP prescribing of ACEIs and ARBs. In a sample of alert firings in which we adjudicated through a chart review that the alert was clinically appropriate and that there was no reason for a PCP to withhold treatment, 75% (42/56) of the alert firings with a “chart closure” hard stop resulted in an ACEI or ARB order [17]. However, this result applied only to a very specific subset of encounters that represented clear opportunities for treatment. This study investigates the broader effects of the BPA with a “chart closure” hard stop by examining all primary care encounters in which an ACEI or ARB appears to be indicated for a patient with diabetes.

### Objectives

The study objective was to evaluate whether the implementation of this BPA alert was associated with changes in ACEI and ARB prescriptions for patients with comorbid diabetes and hypertension across the entire UCLA Health primary care network. We used a quasi-experimental difference-in-differences design with data between 2014 and 2017 to compare the changes in ACEI and ARB prescribing among sites that implemented the BPA during this time frame with the control sites that chose not to implement the BPA during the designated time frame.

## Methods

### Best Practice Advisory With a “Chart Closure” Hard Stop for Comorbid Diabetes and Hypertension Control

UCLA Health implemented the BPA for comorbid diabetes and hypertension control within the context of a pharmacist-led medication management program (MMP) [18] designed to improve medication adherence and cardiovascular risk factor control in primary care. The MMP was rolled out in select primary care sites between 2012 and 2016. MMP pharmacists collaborated with primary care physicians to conduct medication therapy management, provide education to patients, help patients address cost-related issues, conduct medication reconciliation, and correct potential medication problems. In terms of the BPA, the pharmacists provided education to primary care physicians on the alerts and occasionally followed up with those who received alerts. Operational leaders of all primary care sites made two independent decisions: (1) whether to participate in the MMP and (2) whether to implement the BPA.

During a primary care encounter at a BPA implementation site, the BPA fires an alert if the patient meets the following criteria: (1) diabetes diagnosis on the problem list, (2) blood pressure value in the current primary care encounter exceeds 140/90, (3) average blood pressure value from the last three primary care encounters (including the current one) exceeds 140/90, (4) no active ACEI or ARB prescription, (5) no documented allergy or intolerance to both ACEIs and ARBs, (6) age between 18 and 75 years, (7) not pregnant, and (8) no creatinine test before the current primary encounter with a value greater than or equal to 3. In our previous study, we found that the BPA fired alerts in approximately 3% of the encounters for patients with diabetes [17].

**Figure 1:**
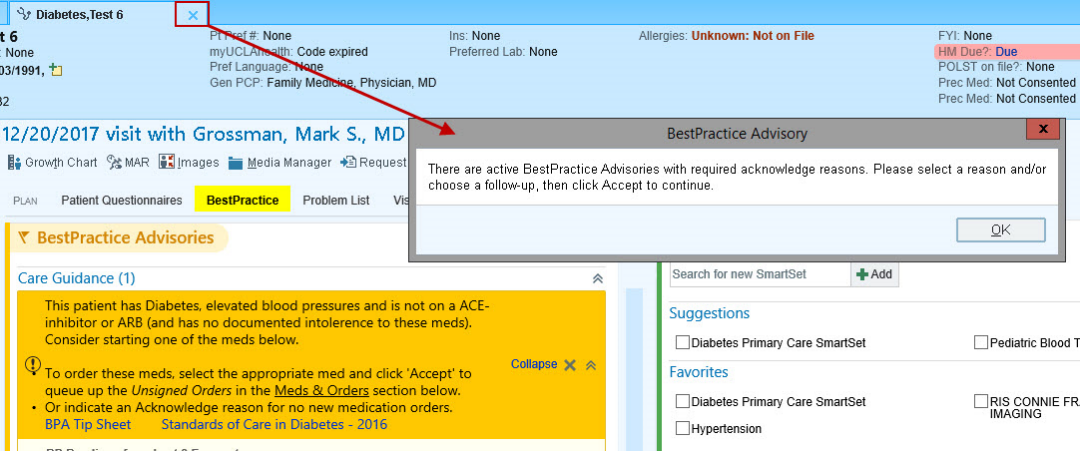
A “chart closure” hard stop prevents primary care providers from closing a patient’s chart without acting on the Best Practice Advisory alert.

**Figure 2:**
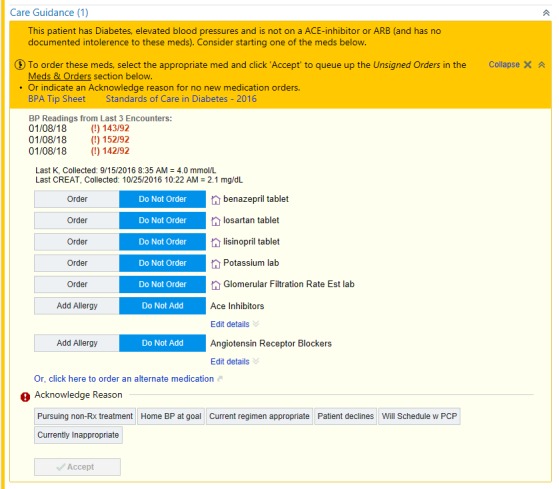
The Best Practice Advisory prompts primary care providers to order an angiotensin-converting enzyme inhibitor or angiotensin-receptor blocker or to dismiss the alert by clicking an acknowledge reason. Home BP at goal: Home blood pressure at goal; Pursuing non-Rx treatment: pursuing nonprescription treatment; Will Schedule w PCP: will schedule with primary care provider.

When the BPA fires an alert, the “chart closure” hard stop prevents PCPs from closing a patient’s chart without responding to the alert (Figure 1) [17]. A PCP can respond by either ordering an ACEI or ARB within the BPA or by dismissing the alert by clicking an acknowledge reason (Figure 2). If a PCP chooses to order an ACEI or ARB outside the BPA, the alert is automatically dismissed and therefore does not require a response, as the data point that caused the alert to fire (ie, no active ACEI or ARB prescription) was modified. As we described in our previous work [17], PCPs can still escape from responding to an alert by modifying the data that caused the alert to fire or if CareConnect automatically logs out of a patient’s chart because of time-out.

If a PCP dismisses an alert by clicking an acknowledge reason, the BPA locks out for the next 30 to 90 days. During a lockout period, the BPA suppresses the alerts to all PCPs even if it determines that the patient has met the criteria to fire an alert. The lockout feature was intended to minimize alert fatigue. The length of the lockout period depends on the acknowledged reason. For example, clicking on “Pursuing non-Rx treatment” locks out the alert for 90 days, whereas “Currently Inappropriate” locks out the alert for 30 days. CareConnect automatically logging out of a patient’s chart because of time-out does not lock out the BPA.

### Best Practice Advisory Implementation at UCLA Health Primary Care Sites

**Figure 3:**
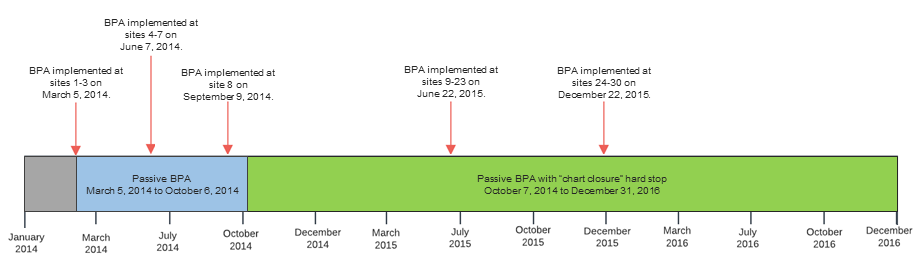
Best Practice Advisory implementation at 30 University of California at Los Angeles Health primary care sites over a 15-month rollout period. The period of interest for this study is from January 2014 to December 2016. BPA: Best Practice Advisory.

A total of 30 primary care sites implemented the BPA over a 15-month rollout period between 2014 and 2015. Figure 3 depicts BPA implementation in relation to the period of interest for this study. In the pilot phase (March 5, 2014, to October 6, 2014), eight sites implemented a passive BPA that did not require a response (ie, ordering an ACEI or ARB within the BPA or dismissing the alert by clicking an acknowledge reason) from PCPs when the BPA fired alerts, but it was found that PCPs rarely responded to these alerts [17]. On October 7, 2014, we added a “chart closure” hard stop to the BPA with the expectation that it would improve PCPs’ visibility of alerts and, therefore, their responses to alerts. Our previous work found that PCP responses to alerts in the eight pilot sites increased significantly from 5.7% (6/105) to 68.2% (122/179) after the addition of the “chart closure” hard stop [17]. Therefore, as of October 7, 2014, all current and future implementation sites used the BPA with the “chart closure” hard stop.

### Study Design

**Figure 4:**
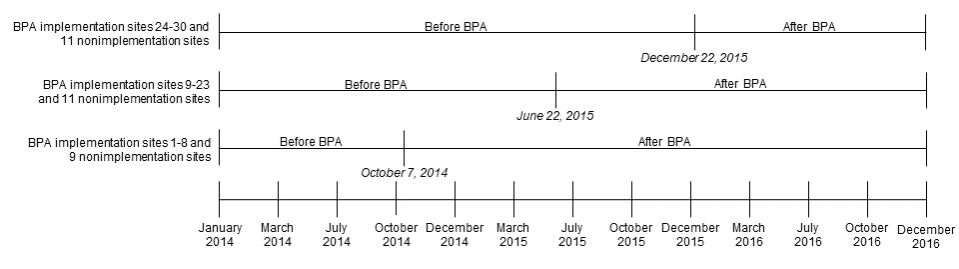
Before and after study periods in the difference-in-differences analysis for the 30 primary care sites that implemented the Best Practice Advisory and the 31 primary care sites that did not implement the Best Practice Advisory. BPA: Best Practice Advisory.

Using a difference-in-differences analysis, we compared changes in ACEI and ARB prescriptions in primary care sites that implemented the BPA (n=30) and sites that did not implement the BPA (n=31) before and after the implementation of the BPA with a “chart closure” hard stop. Our study period was from January 2014 to December 2016. We defined primary care sites at UCLA Health that did not implement the BPA to be nonimplementation (control) sites. As BPA implementation happened over a 15-month rollout period rather than on a single date, we randomly assigned before and after study periods to the 31 nonimplementation sites, which paralleled those of the BPA implementation sites. Figure 4 depicts the before and after study periods in the difference-in-differences analysis for the 30 sites that implemented the BPA and the 31 sites that did not.

### Data Source and Study Sample

We extracted primary care encounter data from CareConnect. The unit of analysis was primary care encounters that represented the opportunities for a PCP to address hypertension among patients with diabetes. To identify these opportunity encounters, we developed an algorithm based on the criteria the BPA uses to fire an alert. The algorithm would enable us to identify opportunity encounters during times in which sites had not implemented the BPA (ie, in BPA implementation sites before BPA implementation and in nonimplementation sites throughout the study period). Similar to the BPA, the algorithm classified a primary care encounter as an opportunity if the patient met the following criteria: (1) diabetes diagnosis on the problem list, (2) blood pressure value in the current primary care encounter exceeded 140/90, (3) average blood pressure value from the last three primary care encounters (including the current one) exceeded 140/90, (4) no active ACEI or ARB prescription, (5) no documented allergy or intolerance to both ACEI and ARB medications, (6) age between 18 and 75 years, (7) not pregnant, and (8) no creatinine test before the current primary encounter with a value greater than or equal to 3. For patients with an opportunity encounter, we extracted data on allergies, diagnoses, laboratory results, medications, problem list, and demographic characteristics. We excluded approximately 5% of the identified opportunity encounters from the analyses because of unknown or missing data on the race or ethnicity of the patients at those encounters.

We also extracted data from CareConnect on BPA alert firings. CareConnect captures in a structured form the date and time of alert firings and PCP response to alerts (ie, ordering an ACEI or ARB within the BPA or dismissing an alert by clicking an acknowledge reason). A limitation of CareConnect is that it does not capture in a structured form whether PCPs escaped from responding to alerts by modifying the data that caused the alert to fire (eg, entering a new blood pressure value that lowers the average or removing diabetes from the problem list), ordering an ACEI or ARB outside the BPA, or being automatically logged out because of time-out.

### Outcome Variable

The outcome was a binary variable indicating whether a PCP ordered an ACEI or ARB on the day of the opportunity encounter or the next day. We used patients’ medication history to construct the variable. For opportunity encounters in which the BPA fired an alert, if a PCP ordered an ACEI or ARB, the variable was considered “ordered” even if the ordering PCP was not the PCP who received the alert. Moreover, the variable was considered “ordered” even if the PCP did not use the BPA to order the prescription.

### Independent Variables

The independent variable of interest was an interaction term for study site (BPA implementation or nonimplementation site) and time (before or after the implementation of BPA with a “chart closure” hard stop). We included as covariates in the adjusted analysis the sex, race, ethnicity, age, blood pressure value at the current encounter, and Charlson Comorbidity Index of patients at the opportunity encounters. We also included in the adjusted analysis a binary variable to indicate whether the site in which the opportunity encounter took place had implemented the MMP at the time of the encounter.

### Main Analysis

We estimated a mixed effects logistic regression model to compare changes in ACEI and ARB prescriptions in opportunity encounters for BPA implementation and nonimplementation sites before and after the implementation of the BPA with a “chart closure” hard stop. We included patient and PCP random effects to account for the clustering of encounters at patient and PCP levels. The estimated coefficient of the interaction term for study site and time provided the difference-in-differences. To describe the difference-in-differences in terms of probability (ie, the change in the probability of an ACEI and ARB prescription from before to after BPA implementation in BPA implementation sites compared with nonimplementation sites), we used predicted probabilities estimated from the regression model.

### Subgroup Analysis

The presence of MMP pharmacists at primary care sites could have increased PCPs’ awareness of the importance of hypertension control in patients with diabetes. Therefore, the MMP may have influenced PCPs’ decisions to prescribe ACEIs and ARBs. For that reason, we conducted a subgroup analysis of opportunity encounters in sites that had implemented the MMP at the time of the encounter vs sites that had not. This enabled us to assess differential changes in ACEI and ARB prescriptions by MMP implementation status. The subgroup analysis used a separate mixed effects logistic regression model than the main analysis. The model for the subgroup analysis excluded observations (ie, patient encounters) in sites that had not implemented the MMP at the time of the encounter.

## Results

### Description of Opportunity Encounters

We identified a total of 2438 opportunity encounters in BPA implementation and nonimplementation sites between January 2014 and December 2016 (Table 1). These opportunity encounters were associated with 1163 unique patients. No patients had opportunity encounters in both BPA implementation and nonimplementation sites.

**Table 1:** Description of opportunity encounters in Best Practice Advisory implementation and nonimplementation sites before and after the implementation of Best Practice Advisory with a “chart closure” hard stop.

Study group	Before BPA^a^			After BPA			Total opportunity encounters, n
	Opportunity encounters, n	Unique patients, n	ACEI^b^ or ARB^c^ ordered, n (%)	Opportunity encounters, n	Unique patients, n	ACEI or ARB ordered, n (%)	
BPA implementation sites	490	249	52 (10.6)^d^	884	392	188 (21.3)^e^	1374
Nonimplementation sites	304	180	38 (12.5)^f^	760	342	92 (12.1)^g^	1064

^a^BPA: Best Practice Advisory.

^b^ACEI: angiotensin-converting enzyme inhibitor.

^c^ARB: angiotensin-receptor blocker.

^d^N=490.

^e^N=884.

^f^N=304.

^g^N=760.

In BPA implementation sites, 72.34% (994/1374) of the opportunity encounters happened in sites that had implemented the MMP at the time of the encounter. In nonimplementation sites, 34.40% (366/1064) of the opportunity encounters happened in sites that had implemented the MMP at the time of the encounter. The difference was statistically significant (*P*<.001).

After the implementation of the BPA with a “chart closure” hard stop, the BPA fired an alert in 72.1% (637/884) of the opportunity encounters in implementation sites. We would not expect an alert firing in 146 of the remaining 247 opportunity encounters with no alert firings as the BPA locked out because of a previous dismissal.

Each patient in our sample had approximately two (2438/1163) opportunity encounters during the study period. Table 2 compares the characteristics of the 1163 unique patients at their first opportunity encounter in BPA implementation and nonimplementation sites. Patients in BPA implementation sites were significantly younger than patients in nonimplementation sites (59.4 years vs 61.4 years; *P*<.001).

**Table 2:** Characteristics of unique patients at their first opportunity encounter, by Best Practice Advisory implementation status.

Patient characteristics		Best Practice Advisory implementation sites (n=641)	Nonimplementation sites (n=522)	*P* value	
Female, n (%)		353 (55.1)	274 (52.5)	.38	
**Race, n (%)**					.99
	White	359 (56.0)	294 (56.3)		
	Black	96 (15.0)	75 (14.4)		
	Asian	74 (11.5)	61 (11.7)		
	Other^a^	112 (17.5)	92 (17.6)		
Latino, n (%)		128 (20.0)	86 (16.5)	.13	
Age (years), mean (SD)		59.4 (0.4)	61.4 (0.4)	<.001	
Systolic blood pressure at the current encounter, mean (SD)		153.8 (0.5)	153.8 (0.5)	.99	
Diastolic blood pressure at the current encounter, mean (SD)		86.4 (0.4)	85.3 (0.5)	.07	
**Charlson Comorbidity Index** **, n (%)**					.11
	1	255 (39.8)	200 (38.3)		
	2	152 (23.7)	103 (19.7)		
	≥3	234 (36.5)	219 (42.0)		

^a^American Indian or Alaska Native, Native Hawaiian or other Pacific Islander, multiple races, and other race.

**Figure 5:**
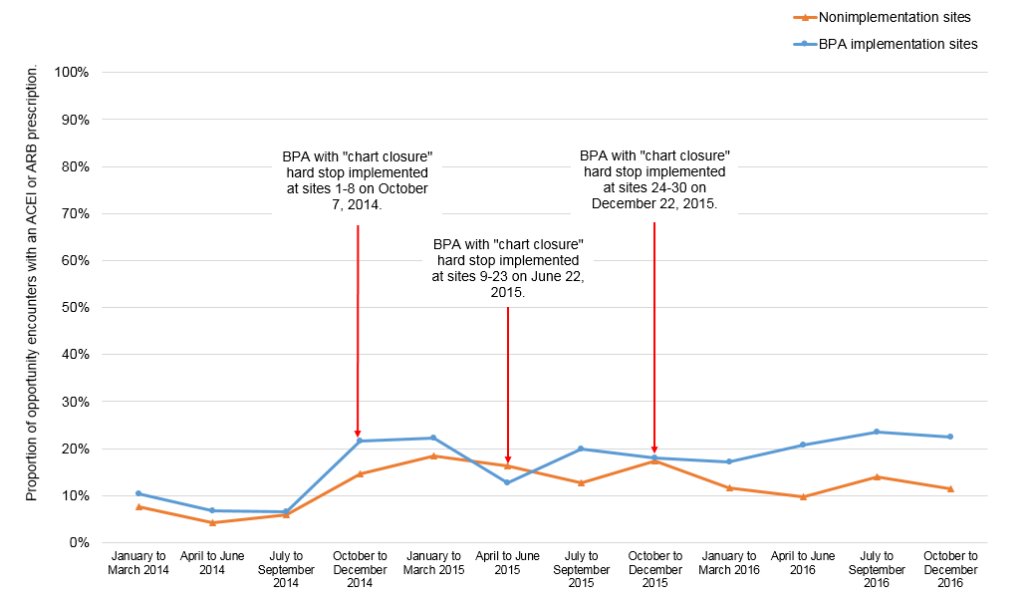
Proportion of opportunity encounters with an angiotensin-converting enzyme inhibitor or angiotensin-receptor blocker prescription in Best Practice Advisory implementation and nonimplementation sites throughout the study period. ACEI: angiotensin-converting enzyme inhibitor; ARB: angiotensin-receptor blocker; BPA: Best Practice Advisory.

Figure 5 plots the proportion of opportunity encounters with an ACEI or ARB prescription in BPA implementation and nonimplementation sites during the study period. Before the first wave of BPA implementation, the trend in the proportion of opportunity encounters with an ACEI or ARB prescription was similar in BPA implementation and nonimplementation sites. At the time of the first wave of BPA implementation (October 2014), the proportion of opportunity encounters with an ACEI or ARB prescription in both study groups increased, although the increase was greater in BPA implementation sites. Over time, as more sites began implementing the BPA, the proportion of opportunity encounters with an ACEI or ARB prescription was generally higher in BPA implementation sites compared with nonimplementation sites.

### Changes in Angiotensin-Converting Enzyme Inhibitor and Angiotensin-Receptor Blocker Prescriptions After Best Practice Advisory Implementation

Table 3 presents the results of the mixed effects logistic regression analysis on ACEI and ARB prescriptions during opportunity encounters. The interaction term for study site (BPA implementation or nonimplementation site) and time (before or after the implementation of BPA with a “chart closure” hard stop) was statistically significant. This indicates that the change in prescriptions before implementation vs after implementation was significantly greater in BPA implementation sites than in nonimplementation sites.

Table 4 presents the difference-in-differences estimate for the predicted probability of an ACEI or ARB prescription during an opportunity encounter. The predicted probability of a prescription increased from 11.46% to 22.17% during opportunity encounters in BPA implementation sites after BPA implementation (*P*<.001). The predicted probability of a prescription decreased from 16.16% to 15.04% during opportunity encounters in non-BPA implementation sites, although the change was not statistically significant. Overall, the change in the predicted probability of an ACEI or ARB prescription from before to after BPA implementation was significantly greater in BPA implementation sites compared with nonimplementation sites (difference-in-differences of 11.82; *P*=.001).

**Table 3:** A mixed effects logistic regression analysis on angiotensin-converting enzyme inhibitor or angiotensin-receptor blocker prescribing in response to opportunity encounters.

Variable			Exponential (coefficient)	*P* value	95% CI
**BPA^a^ implementation characteristics**					
	BPA implementation site^b^		0.58	.13	0.29 to 1.17
	Post BPA implementation		0.89	.68	0.51 to 1.56
	BPA implementation site×post BPA implementation		3.34	.001	1.59 to 7.02
**Patient characteristics**					
	Female		0.62	.01	0.44 to 0.88
	**Race**				
		Black	1.14	.61	0.69 to 1.87
		Asian	2.18	.01	1.27 to 3.73
		Other^c^	1.30	.25	0.83 to 2.04
	Latino		1.03	.91	0.66 to 1.59
	Age (years)		0.99	.54	0.98 to 1.01
	Current systolic blood pressure		1.02	<.001	1.01 to 1.03
	Current diastolic blood pressure		1.02	.01	1.01 to 1.04
	**Charlson Comorbidity Index**				
		2	0.62	.03	0.40 to 0.96
		≥3	0.51	.001	0.35 to 0.76
Post medication management program implementation			1.85	.01	1.20 to 2.85

^a^BPA: Best Practice Advisory.

^b^No patients had opportunity encounters in both Best Practice Advisory implementation and nonimplementation sites.

^c^American Indian or Alaska Native, Native Hawaiian or other Pacific Islander, multiple races, and other race.

**Table 4:** Changes in angiotensin-converting enzyme inhibitor and angiotensin-receptor blocker prescriptions before vs after the implementation of Best Practice Advisory with a “chart closure” hard stop.

Predicted probability of an angiotensin-converting enzyme inhibitor or angiotensin-receptor blocker prescription during an opportunity encounter^a^	Before Best Practice Advisory	After Best Practice Advisory	Difference	*P* value
Best Practice Advisory implementation sites, %	11.46	22.17	10.70	<.001
Nonimplementation sites, %	16.16	15.04	−1.12	.69
Difference-in-differences (95% CI)	N/A^b^	N/A	11.82 (0.05 to 18.7)	.001

^a^We adjusted the mixed effects logistic regression model for sex, race, ethnicity, age, current blood pressure, Charlson Comorbidity Index, and whether the primary care site in which the opportunity encounter took place had medication management program at the time of the encounter, as well as patient and primary care provider random effects to account for clustering of encounters at the patient and provider levels.

^b^N/A: not applicable.

### Subgroup Analysis

Table 5 presents the difference-in-differences estimate for the predicted probability of an ACEI or ARB prescription during an opportunity encounter, by MMP implementation status. When the MMP had been implemented at the time of the encounter, the change in the predicted probability of an ACEI or ARB prescription from before to after BPA implementation was significantly greater in BPA implementation sites compared with nonimplementation sites (difference-in-differences of 25.41; *P*<.001). The large difference-in-differences was driven by a significant increase in the probability of a prescription in BPA implementation sites coupled with a significant decrease in the probability of a prescription in nonimplementation sites. Conversely, when the MMP had not been implemented at the time of the encounter, the change in the predicted probability of an ACEI or ARB prescription from before to after BPA implementation was not significantly different in the two study groups (difference-in-differences of 1.58; *P*=.74).

**Table 5:** Changes in angiotensin-converting enzyme inhibitor and angiotensin-receptor blocker prescriptions before vs after the implementation of Best Practice Advisory with a “chart closure” hard stop, by MMP implementation status.

Predicted probability of an angiotensin-converting enzyme inhibitor or angiotensin-receptor blocker prescription during an opportunity encounter^a^		Before BPA^b^	After BPA	Difference	*P* value
**MMP^c^ implemented**					
	BPA implementation sites, %	11.07	25.38	14.31	<.001
	Nonimplementation sites, %	25.83	14.73	−11.10	.03
	Difference-in-differences (95% CI)	N/A^d^	N/A	25.41 (14.05 to 36.77)	<.001
**MMP not implemented**					
	BPA implementation sites, %	10.36	16.37	6.01	.11
	Nonimplementation sites, %	8.69	13.11	4.42	.13
	Difference-in-differences (95% CI)	N/A	N/A	1.58 (−7.78 to 10.94)	.74

^a^We adjusted the mixed effects logistic regression model for sex, race, ethnicity, age, current blood pressure, and Charlson Comorbidity Index, as well as patient and primary care provider random effects to account for clustering of encounters at the patient and provider levels. The site in which the opportunity encounter took place either did or did not have the medication management program at the time of the encounter.

^b^BPA: Best Practice Advisory.

^c^MMP: medication management program.

^d^N/A: not applicable.

## Discussion

### Principal Findings

In this study, using a quasi-experimental difference-in-differences design, we found that patient encounters at UCLA Health primary care sites that implemented the BPA with a “chart closure” hard stop were significantly more likely to result in an ACEI or ARB prescription for patients with diabetes compared with encounters in nonimplementation sites. However, in a subgroup analysis, we found that only BPA implementation sites that had also implemented the MMP experienced significant improvements in ACEI and ARB prescribing. These conclusions are based on the following evidence. First, our findings reveal that, overall, BPA implementation nearly doubled the probability of a PCP ordering the indicated ACEI or ARB prescription after BPA implementation, compared with no significant change in this probability in nonimplementation sites over the same study period (Table 4). Second, BPA implementation coupled with the MMP more than doubled the probability of a PCP ordering an ACEI or ARB (Table 5). In contrast, there was no significant improvement in this probability in BPA implementation sites without the MMP. Collectively, this evidence supports the concept that a BPA with a “chart closure” hard stop, a feature intended to reduce disruption to PCP workflow, is a promising CDS tool for the treatment of patients, especially when implemented within the context of multidisciplinary, team-based care, in which clinical pharmacists support the work of PCPs [18].

### Comparison With Previous Work

Our previous study examined patient encounters with an alert firing between March 2014 and October 2014 in the initial eight sites that implemented the BPA [17]. We found that PCPs rarely responded (ie, ordered an ACEI or ARB within the BPA or dismissed the alert by clicking an acknowledge reason; 94% of the alert firings had no response) when the BPA fired passive alerts. Although it is common for PCPs to ignore or override CDS alerts [19,20], the PCPs in our study indicated that they simply did not notice the passive alerts. Others have also observed that passive, noninterruptive alerts to providers have low visibility [21]. After the addition of the “chart closure” hard stop to remedy the issue, PCPs responded to alert firings more often (only 20%-27% of the alert firings had no response). However, PCPs’ main response was to dismiss the alerts rather than to order an ACEI or ARB. Thus, even when PCPs noticed the alert, they chose to ignore the alert’s recommendations, which suggests that PCPs may not have trusted the BPA in the early stages of implementation. On the basis of the results of this study, which examines the impact of the alert over a longer post period, we posit that, over time, PCPs began trusting the BPA. PCPs’ trust in the BPA may have developed with help from the MMP that was implemented in some of the clinics, where it would be likely for pharmacists to explain to PCPs all the considerations that went into the alert’s recommendation. This, coupled with the “chart closure” hard stop, which PCPs learned would stop them from closing a patient’s chart without acting on the alert, may be an indication that PCPs changed their attitude toward the BPA and thus began prescribing ACEIs and ARBs rather than simply dismissing the alerts. Future qualitative research is needed to explore these assertions.

This study found that when the MMP had been implemented in primary care sites at the time of the opportunity encounter but the BPA had not been implemented because of the operational leaders’ decision not to participate, the predicted probability of an ACEI or ARB prescription was significantly lower in the period after the BPA had been implemented at other sites. This suggests that PCPs practicing at sites with MMP pharmacists but without the BPA were less likely to prescribe an ACEI or ARB when there was an opportunity. A possible explanation for this observation is that PCPs may have been increasingly relying on MMP pharmacists to take responsibility for patients. Thus, over time, the PCPs may have attended less to opportunities to prescribe an ACEI or ARB to the patient.

Complementary to the findings of this study, previous research has found that the implementation of EHR-based CDS tools can improve process outcomes for diabetes care. O’Conner et al [22] studied a CDS tool (the “Diabetes Wizard”) that, among other features, could suggest to PCPs specific medications for patients with elevated blood pressures at the current encounter. In a randomized trial, they observed a small improvement in the proportion of patient encounters with blood pressure measurements in the CDS intervention group before vs after the intervention compared with a control group. PCPs reported intensifying blood pressure treatment in 43.6% of the encounters with patients with diabetes and elevated blood pressure, although treatment intensification could include the use of antihypertensive medications or of lifestyle interventions. Other randomized trials of EHR-based CDS tools have reported improvements in additional process-related outcomes for diabetes care, including increased hemoglobin A_1c_ and cholesterol testing [22-26].

In contrast to our findings that the BPA with a “chart closure” hard stop was associated with improvements in PCPs ordering an ACEI or ARB, Schnipper et al [26] found that a smart form documentation tool with CDS capability was not associated with improvements in ACEI and ARB prescribing for patients with diabetes. However, in the CDS tool that Schnipper et al [26] studied, PCPs had to initiate the use of the smart form during patient encounters. Schnipper et al [26] found that PCPs chose to use the tool in fewer than 4% of the eligible patient encounters. Conversely, the use of the BPA in this study did not depend on PCPs changing their usual EHR workflow, as the alerts were fully integrated within the existing workflow. Similarly, O’Connor et al [22] did not find significant improvements in new prescriptions of antihypertensive medications for patients with diabetes and elevated blood pressure. Unlike our BPA, which fired if patients had elevated blood pressures over multiple encounters, O’Connor et al’s [22] “Diabetes Wizard” would suggest an antihypertensive treatment based only on the blood pressure value at the current encounter. In the latter case, PCPs might be less willing to prescribe antihypertensive medications based on a single blood pressure elevation, especially if patients’ previous documented blood pressure values were in the recommended range.

BPAs are commonly used for electronic CDS in primary care [12-14]. However, the impact of BPAs on ACEI and ARB prescribing for patients with diabetes and elevated blood pressures has not been reported [3]. BPAs with “chart closure” hard stops, which fire passive alerts and wait until the end of an encounter to force an action, are intended to get PCPs’ attention without excessively disrupting their workflow. This study showed that a BPA alert with a “chart closure” hard stop had a modest but statistically significant effect (from 11% to 22%) on improving prescribers’ responses to overlooked opportunities for improved diabetes care. Our previous work showed that the “chart closure” hard stop succeeded in getting the BPA noticed [17], but an obvious disadvantage is that the BPA may be noticed after the patient has left the office, when it is less convenient to discuss starting a new medication. Prescribers tended to be more vigilant and to act more immediately on the BPA both over time (in the latter months, as they learned that they could not escape responding to the alert) and if they were in the subgroup with the pharmacist-led program in their practice.

### Limitations

This study has several limitations. First, the study was not randomized; instead, operational leaders at the various sites made the decision of whether to implement the BPA. We found some systematic differences in the characteristics between the BPA implementation and nonimplementation sites that are related to the outcome, but using the statistical methods of quasi-experimental study design, we controlled for these characteristics, and our differences-in-differences estimate should be unbiased. Second, we cannot exclude the possibility that a contemporaneous but unrelated event in either group of primary care sites confounded the results. Third, the BPA did not fire an alert in about 28% of the opportunity encounters that we identified in BPA implementation sites after BPA implementation, largely because of “lockouts” after previous dismissals. To the extent that these dismissals were truly appropriate, we identified some opportunities erroneously, which would bias our results toward the null. However, to the extent that the alert failed to fire for true opportunities, our results reflect the true shortcomings of the alert as implemented. In our previous study, after reviewing patient charts associated with the 284 alerts that fired during the pilot phase implementation, we judged 37.7% (107/284) of the alert firings to be unnecessary or inappropriate [17]. We deemed the remaining 62.3% (177/284) of the alert firings to be clinically appropriate. Thus, based on the findings from our previous study, we would expect that about 62% of the opportunity encounters identified in this study actually represent true opportunities to prescribe an ACEI or ARB.

### Conclusions

Overall, we found that primary care encounters in sites that implemented a BPA with a “chart closure” hard stop to notify PCPs of the opportunities to treat hypertension in patients with diabetes were more likely than control sites to result in an ACEI or ARB prescription. However, we only observed a significant improvement in ACEI and ARB prescribing in the subset of BPA implementation sites that had also implemented the MMP at the time of the encounter. This study’s findings contribute new knowledge on the impact of BPAs on ACEI and ARB prescribing for patients with diabetes. They also shed light on the potential benefits of using “chart closure” hard stops, which are intended to minimize PCPs’ workflow disruption, although future research is needed to gain a better understanding of the user experience.

## Abbreviations

ACEI: angiotensin-converting enzyme inhibitor
ARB: angiotensin-receptor blocker
BPA: Best Practice Advisory
CDS: clinical decision support
EHR: electronic health record
MMP: medication management program
NIA: National Institute on Aging
PCP: primary care provider
UCLA: University of California at Los Angeles
